# Leveraging a clinical research information system to assist biospecimen data and workflow management: a hybrid approach

**DOI:** 10.1186/2043-9113-1-22

**Published:** 2011-08-25

**Authors:** Prakash M Nadkarni, Rowena Kemp, Chirag R Parikh

**Affiliations:** 1 Yale University School of Medicine, New Haven, CT, USA; 2 Clinical Epidemiology Research Center, VAMC, West Haven, CT, USA

**Keywords:** Biospecimen data management, clinical research information systems, multi-center clinical studies, biorepositories

## Abstract

**Background:**

Large multi-center clinical studies often involve the collection and analysis of biological samples. It is necessary to ensure timely, complete and accurate recording of analytical results and associated phenotypic and clinical information. The TRIBE-AKI Consortium http://www.yale.edu/tribeaki supports a network of multiple related studies and sample biorepository, thus allowing researchers to take advantage of a larger specimen collection than they might have at an individual institution.

**Description:**

We describe a biospecimen data management system (BDMS) that supports TRIBE-AKI and is intended for multi-center collaborative clinical studies that involve shipment of biospecimens between sites. This system works in conjunction with a clinical research information system (CRIS) that stores the clinical data associated with the biospecimens, along with other patient-related parameters. Inter-operation between the two systems is mediated by an interactively invoked suite of Web Services, as well as by batch code. We discuss various challenges involved in integration.

**Conclusions:**

Our experience indicates that an approach that emphasizes inter-operability is reasonably optimal in allowing each system to be utilized for the tasks for which it is best suited.

## 1 Background

Research to improve health care is increasingly supported by advances in genomics, proteomics and metabolomics. To allow statistically meaningful analyses, all of these methodologies demand large numbers of adequately collected and annotated biospecimens from both diseased and non-diseased individuals [1], which can often be obtained only through multi-center studies. It is essential to ensure timely, complete and accurate recording of analytical results and associated phenotypic and clinical information. Well-managed Biorepositories - entities that support receipt, storage, processing and/or distribution of biospecimens [2] through standardized operating procedures, along with management of their associated data- have consequently become essential aids in investigating the causes and prognosis of human diseases.

Development of biomarkers for acute kidney injury (AKI) is a top research priority: the US National Institute of Diabetes, Digestive and Kidney Diseases, part of the NIH, supports the TRIBE-AKI consortium (Translational Research Investigating Biomarker Endpoints in Acute Kidney Injury) for this purpose http://www.yale.edu/tribeaki/. AKI occurs in 2-5% of hospitalized patients - it complicates shock due to any cause, trauma with muscle injury, hemolytic conditions and cardiac surgery, among other conditions [3]. Outcomes associated with AKI have remained unchanged over several decades, and large multi-center studies may be necessary to ensure adequate cohort/sample size for various purposes, e.g., biomarker development and validation.

Multi-center studies often involve biospecimen collection at various sites and shipping of biospecimens between sites and a sample coordinating center for purposes of storage and analysis. Related informatics support involves tasks such as barcode generation, biospecimen storage/inventory management, tracking of biospecimen requests and aliquot consumption, and management of the analytic data generated from the specimens. Organizations such as the International Society for Biological and Environmental Repositories (ISBER) provide guidelines and best practice suggestions for standard operating procedures to create and operate a Biorepository, e.g., [2,4]. Most of the guidelines, however, focus on biospecimen banking and distribution, and not on data management [5].

This paper describes the design and implementation of a biospecimen data management system (BDMS), originally developed for the TRIBE-AKI consortium, that facilitates the workflow involved in multi-centric scenarios that involve longitudinal cohort follow-up with biospecimen collection and analysis. The system also communicates bi-directionally with a clinical research information system (CRIS) that manages the analytic data.

## 2 Construction and Content

To provide a rationale for our architectural decision, we first describe multi-centric study workflow, which dictates software requirements and design. We then summarize the issues of overlapping functionality between BDMS and CRIS software, and user interfaces to clinical/biospecimen data.

### 2.1 Workflow of Biospecimen Collection and Processing in Multi-centric studies

Enrollment of patients based on the protocol's inclusion and exclusion criteria is a complex process as such individuals are rarely available immediately. The study protocol's "e*vent calendar*", a predetermined sequence of time points ("events") relative to a subject's enrollment date, determines the biospecimen-collection schedule. Note that many or even most time-points are not associated with biospecimen collection, but may involve subject interviews, clinical examination, special investigations (e.g., radiology) or outreach (e.g., reminders through phone, letters or E-mail). The numerous study parameters recorded across all events, such as measures of disease progression or clinical improvement specific to the disease condition being followed, are segregated into logically-related units called *case report forms *(CRFs).

In order to reduce shipping costs, centers perform local biospecimen processing, aliquot creation and temporary storage prior to batch shipments. The actual number of aliquots may vary for individual subjects because of material-collection constraints (especially in pediatric patients): in intensive-care/emergency situations, scheduled collections may be missed. Actual biospecimen collection and quantity must be closely tracked to inform the study progress. To streamline collection and processing, an analytic center typically provides collection centers in advance with a batch of aliquot containers (vials) and the barcode labels record standard information such as patient ID, event, sample type and aliquot number.

The samples are batch shipped and aliquots that are received are scanned at the data and sample coordinating center for verification against the previously entered collection data. Discrepancy-resolution generally involves human intervention (e.g., phone calls to collection centers). After any additional local processing if necessary, aliquots are stored in freezers, with locations recorded using a coordinate system (e.g., site-freezer-rack-slot). Biospecimens are consumed following local analysis or shipping to external biomarker laboratories, either in bulk for specialized analyses, or when individually requested by collaborators. For the former, the external lab may send analytical results back in a variety of formats (typically in spreadsheets), and these must also be bulk-imported. Specimen consumption must be tracked accurately to guide future ancillary studies and sample requests.

### 2.2 Existing Software for Biospecimen Management

Because individual research groups' needs vary greatly, existing BDMS functionality is very diverse: however, all BDMSs should be able to manage an unlimited number of study protocols: every data element must be associated, directly or indirectly, with the study where it originated.

Angelow et al [6] describe a "virtual repository" BDMS: biospecimens are not shipped, but stored (and analyzed) at individual collection centers, but managed by a central web-based BDMS. Pulley et al [7] describe a DNA biobanking system for anonymous subjects: each biospecimen is associated with structured and textual electronic-medical-record (EMR) data that is anonymized using electronic and manual processes. This data characterizes individual phenotypes: genotype-phenotype correlations form a focus of the eMERGE network [8].

CaTissue [9], supported by the Cancer BioInformatics Grid (CaBiG) [10], focuses on tissue banking, providing functionality such as clinical annotations (e.g., pathology reports), but also has general-purpose features. The annotation module has been utilized by other groups [11,12].

### 2.3 CRISs and BDMSs: Overlapping Functionality

Clinical Research Information Systems (CRISs) [13-15], with prices ranging from free to several million dollars, are designed to manage workflow and data for an arbitrary number of studies. Both CRISs and BDMSs typically utilize high-end relational database management systems (RDBMSs). When BDMSs are used for clinical studies, they address many areas covered by CRISs (though often in greater depth) as discussed shortly. Despite this overlap, even high-end CRISs do not currently provide comprehensive BDMS capability: biospecimen-inventory management, in particular, falls significantly short.

Large research groups therefore employ both types of systems. In such scenarios, one must determine whether one system shall be used primarily for a particular function (or whether both should be used for complementary functionality), and how to coordinate both systems' contents. Consider the following synchronization challenges:

1. Users: A large multi-center study may involve hundreds of research staff across sites, with a variety of access privileges to either system: staff turnover may be significant. We consider this issue later in the Discussion.

2. Informed Consent: Consent often has finer details related to the degree of participation allowed by the subject. Based on research goals, subjects may consent to provide some tissues but not others, or to have only certain tests performed: e.g., they may decline genotyping because of concerns (in the USA) that accidental result disclosure may impact their families' health-insurability. Biospecimens may inherit their consent values from the subject (e.g., if the subject drops out and withdraws consent, the consent status of all specimens must automatically change).

3. Collection Schedules: As stated earlier, the study calendar is a *superset *of the biospecimen-collection calendar. For subjects' convenience, individual collection visits also serve other purposes (e.g., physical examination, interviews), and visits are frequently rescheduled.

4. Analytical Data: The subject's total clinical data constitute a superset of biospecimen-associated analytic data, which are rarely inspected in isolation. Research staffs typically enter/edit non-analytical data, either through real-time electronic data capture, or on paper that is later transcribed electronically by data-entry staff. While analytical data can also be entered manually, many parameters may be outputted electronically by laboratory instruments following batch analyses, and are preferably bulk-imported.

When both systems are in use, issues 3-4 above result in maximizing CRIS use. However, there is some data overlap - e.g., patient identifiers, basic study protocol information, etc. and consequently, data exchange is unavoidable.

### 2.4 User Interfaces for Clinical Data

User interfaces for interactive data capture must support robust validation and ergonomics. Parameter-level validation includes data type, range and set-membership, and mandatory (non-empty) values. Cross-parameter validation involves testing of rules (e.g., the differential white blood cell count components must total 100). Ergonomic aids include automatic computations of parameters based on formulas, disabling of certain fields based on values of previously entered fields (so called "skip logic") and keyword-based search of controlled biomedical vocabularies. Finally, based on the study calendar, individual parameters may only be recorded for the CRFs/time-points where they apply. The approach of programming such capabilities manually (e.g., Angelow et al). takes significant expertise and effort, and does not scale. Alternative user-interface-management approaches include:

1. Managing collection schedules and analytical data through the BDMS. CaTissue lets developers specify a Unified Modeling Language (UML) data model, generating relational tables and a basic form interface that supports only data-type and set-membership checks. Calendar functionality (e.g., reminders, reports) lags considerably behind that of CRISs,.

Several commercial BDMSs (e.g., FreezerPro [16] and FreezerWorks [17]) provide more end-user-friendly and more full featured alternatives: some of these are Web-based, while others use two-tier technology (i.e., custom client software installed on multiple desktops communicating directly with a database). In any case, such systems address longitudinal-clinical-study needs only partially.

2. Delegating calendar and analytical-data management to a CRIS. CRISs typically provide extensive interface-generation as well as calendar-driven capabilities: they allow designer-level users to specify the interface declaratively through a data library, and then generate CRFs. We employ this design approach.

## 3. System Architecture

The BDMS communicates bi-directionally with a full-function Web-based open-source CRIS, TrialDB [18,19], which has the ability to generate full-featured CRFs. TrialDB is a general-purpose CRIS that has been used for studies ranging from psychiatry, medical and surgical oncology to endocrinology. The CRIS is also the BDMS's external face. In our current set-up, only a few individuals, limited to a single laboratory, need edit access to the BDMS: external users need read-only access to subsets or aggregates of the BDMS data. The limited-edit-access constraint allows us to implement the BDMS using an Intranet-access-only, two-tier design - a Microsoft Access front-end to a Microsoft SQL Server RDBMS.

Two-tier solutions are inherently less scalable than Web-based ones, which are "three-tier" - a Web-server application intervenes between the client (browser) and the database. However, greater toolset maturity allows significantly easier software development and modification, which is important when the system's functionality is evolving rapidly. Also, we use code libraries to facilitate eventual porting to a Web-based architecture (as discussed later): TrialDB itself was developed this way.

### 3.1 Database Schema

**Figure 1:**
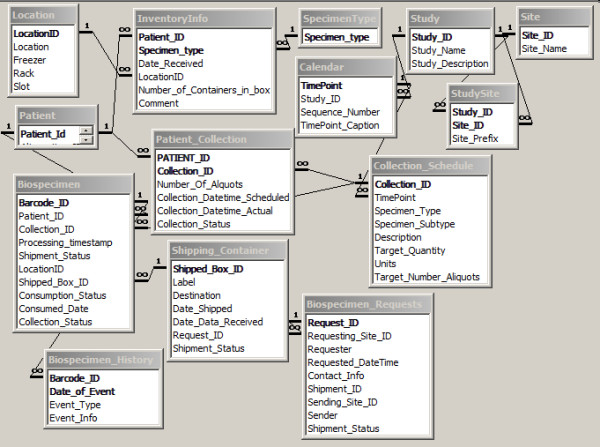
**Database schema**.

Figure 1 illustrates the database schema. Additional File 1 contains an annotated description of individual tables and columns.

The tables can be grouped into the following categories:

1. Metadata (definition) tables imported from the CRIS: these contain a subset of the corresponding CRIS information - the bare minimum necessary for the BDMS to function. Thus we have basic information on study protocols, research sites, types of specimens, calendar information, and the planned collection schedule (including the number of specimens/aliquots of each type scheduled for collection at each time-point).

Metadata is imported after study-protocol definition. It changes very infrequently during the study (significant changes to the protocol typically have to be IRB-approved): BDMS-CRIS synchronization typically happens just once.

2. Subject/patient-related data imported from the CRIS. This data (also a CRIS subset) includes basic patient-identifying information and enrollment status, plus information on the specimens/aliquots actually collected. Synchronization is periodic - just before anticipated arrival of a sample batch, or when certain changes occur in the CRIS.

3. Biospecimen/Inventory data managed primarily by the BDMS: available storage locations, actual storage locations for specimens, details of individual biospecimens, shipping requests and shipments, and a history of operations performed on a biospecimen (e.g., shipping, processing, consumption).

4. Mapping Tables: (not shown in figure). These tables, which record the correspondence between BDMS and CRIS data elements, facilitate export of BDMS data to the CRIS. These tables have a structure highly specific to the CRIS, and are not discussed further.

### 3.2 System Functionality

We summarize BDMS functionality under the following categories:

• Barcode generation: Barcode labels for each aliquot container are generated (using the Abarcode Inc. toolset, http://www.abarcode.net) according to designer-specified templates: e.g., in addition to a machine generated barcode with a check-digit, we also include identifying information such as surrogate Patient ID, collection date, protocol ID and specimen type. Based on collection circumstances, all aliquot containers may not be utilized.

Barcodes are stored as strings rather than numbers: this allows database pattern-match search in the uncommon (but not negligible) event of partial scan.

• Inventory/storage: The capability includes: assign storage location for specimens, locate a given specimen, all specimens for a given patient or set of patients, summarize contents of a given location/sub-location, list unused locations, track sample consumption, report available aliquots for a given subject/time-point, etc.

• Shipping Management: Functions include: accept new specimens, select multiple samples for external shipping/analysis, list specimens associated with a given shipping container, etc.

• Bulk Import of analytical results into CRIS: Results arrive in a variety of data formats, e.g., Excel spreadsheets. Rather than force external labs to return data in a specific format, we accept their format and bulk-import data using a set of mapping tables that map columns in their data (patient ID, time-point, analytical result) to CRIS data elements. Mapping is performed through a point-and-click interface. Utilization of specimen aliquots by analytical processes is also used to track consumption and update inventory. Similarly, we can track requests associated with individual patients (typically made by research collaborators).

• Consent Management: We do not try to manage specimen-consents within the BDMS: these are simulated in the CRIS by treating different types of consent as though they were clinical parameters. We have found this approach workable.

### 3.3 Integration between CRIS and BDMS

There are two types of situations where synchronization of CRIS and BDMS are needed.

#### 3.3.1 Interactive Updates

These typically involve a single subject, and mostly occur when an end-user is interacting with the CRIS using a CRF; a real-time push of data related to that subject from the CRIS, or a pull from the BDMS, is needed. Inter-system communication occurs through a Web service implemented using the lightweight REST (Representational State Transfer) approach [20]. Here, the client (i.e., the Web page) communicates with a server through a uniform interface consisting of a series of self-descriptive messages. No client context is stored on the server between requests: i.e., the invocation is stateless.

An extension mechanism built into TrialDB allows a service specification to be part of the CRF definition: the specification consists of the service URL (which is https-based), a caption and description. When the CRF is generated, a button with the caption (and an accompanying description/explanation) is created at the foot of the page. Clicking the button executes the URL, which takes a single parameter, the symmetrically encrypted primary-key value of the CRF instance in the CRIS. This value allows the service to determine the current Subject, Study/Protocol, TimePoint, and the values of individual clinical parameters embedded within the current CRF.

In the case of the CRIS, the service is implemented part of the CRIS application, so that it is able to utilize the current session information (which records information such as the current user, current study that the user is working with, etc.) for authentication. Effectively, an additional parameter, a uniquely identifiable session ID, is passed in the URL by the Microsoft ASP.NET framework (which is used to create the Web application). The service accesses both the BDMS and CRIS database schemas directly using the well-known Open Database Connectivity (ODBC) protocol [21], which allows programmatic access to diverse RDBMSs using a vendor-independent SQL syntax.

#### 3.3.2 Batch Updates

Batch operations typically push summarized BDMS data of multiple patients- e.g., number of currently available biospecimens/aliquots for all subjects (by time-point and specimen type) - into the CRIS. Here, the BDMS front-end code accesses both schemas using ODBC directly. Here, a REST approach is possible (Microsoft Access supports Web service invocation), but it is probably overkill currently. However, we do not rule it out if the BDMS concurrency load increases in the future.

## 4. Utility and Discussion

### 4.1 The Challenges of Creating "Universal" BDMSs

It is challenging to create BDMSs to meet all possible purposes equally well. While CaTissue aims to be general-purpose, it has the following limitations.

• As previously stated, analytical-data-interface-design and calendar capabilities fall well short of standard CRIS functionality.

• Biospecimen-related workflow is excessively elaborate for most clinical studies, which limit biospecimens to simpler tissue sources (e.g., blood, urine, DNA).

• Barcode-generation functionality that is built into most BDMSs must be programmed by creating a Java-based Web service.

• It lacks biospecimen-lineage-tracing functionality: in combination with storage-location information, this helps identify possible contamination, which occurred with HeLa cells [22,23].

• The CaTissue data-security model does not address subjects' *Personal Health Information *(PHI). PHI must typically be stored encrypted in multi-centric studies where subjects have not consented to have their PHI accessible outside their own site. (Angelow et al implement site-specific PHI encryption, with dynamic decryption within https for web-based viewing.) Plaintext-PHI-storage increases the risks of accidental/malicious disclosure, as happened with the Epsilon break-in [24].

CaTissue attempts to handle privacy by making PHI columns optional. This strategy, unfortunately, makes the software unusable for operations involving interaction with subjects (for scheduling, or personal follow-up). To prevent patient- misidentification errors in clinical care, WHO guidelines [25] require patient-identity confirmation using least 2 PHI identifiers, such as name and date of birth - which must also be stored securely. Patients identified within a system only by anonymous alphanumeric IDs have a significant likelihood of misidentification, and are put at risk if analytical results determine clinical interventions or workflow decisions. Therefore it is desirable to establish the right balance between patient privacy and patient safety.

TrialDB uses fairly well-known strategies based on disk-based encryption, combined with role-based access, so that only those individuals who need to see PHI are given access to it. The implementation utilizes dynamic interface generation with suppression of PHI fields as needed. PHI-privileged individuals are typically restricted to data (not just PHI) for subjects from their own site.

A relatively minimalist solution where a CRIS interoperates with a BDMS can be workable because it lets each system focus on what it does best.

### 4.2 Current Status and Future Directions

While TrialDB has been in production use at Yale and elsewhere for at least a decade, the integrated BDMS functionality has been implemented relatively recently, and is in use for four multi-center studies. Our choice of TrialDB was dictated, of course, by our intimate familiarity with it. In theory, we could have extended TrialDB to incorporate BDMS functionality. However, the first version of the BDMS had to be created under somewhat stringent time constraints that, combined with the fortunate requirement of limited edit access, more or less dictated the two-tier development route.

Such a situation is not likely to hold forever, and at some future point, the number of concurrent BDMS users will increase, requiring migration to a Web-based architecture. However, the creation of a separate BDMS has allowed us to iteratively refine it without impacting the stability of the TrialDB code. It also occurred to us that such an approach could serve as a demonstration of interoperation between systems that are likely to evolve independently, so that our architecture could be employed in other institutions that do not have the luxury of being able to modify their CRIS's source code.

### 4.3 Integration Challenges

Consortiums such as CDISC (Clinical Data Interchange Standards Consortium) http://www.cdisc.org are working to facilitate the interchange of data and metadata between CRISs through interchange models such as CDISC-ODM (Operational Data Model) [26]. However, the area of biospecimen collection adds an extra dimension to the problem, which CDISC is not currently addressing. Data interchange between BDMSs, or between CRISs and BDMSs, is therefore likely to require *ad hoc *approaches for a while.

The difficulty of implementing interoperability between systems is greatly magnified by proprietary software with closed architecture or poorly documented internals. Even with open-source, well-documented systems, however, the issue of synchronizing the contents of the systems for overlapping functionality remains. Further, CRISs and BDMSs are not the only two systems involved in clinical study workflow: financial/accounting systems must track the services recorded as performed in the CRIS/BDMS, grants-management software and possibly special-purpose patient-scheduling software must similarly integrate.

We now consider in depth synchronization challenges related to users. Currently, because of the restricted access to the BDMS in our setup, we have not had to deal with this issue, but we expect to be forced to in future.

#### 4.3.1 Managing and Coordinating User Roles across Systems

High-end database applications prohibit database-login access by end-users. Instead, users can only login to the application, which then connects to the RDBMS using a service account. This approach is highly scalable. Most users' interactions with the application consist of browsing and editing operations: modern CPUs, which perform many operations in under a nanosecond, would spend a relative eternity waiting for user actions. A single service account can multiplex to serve numerous users, connecting to the database only for the few milliseconds needed to fulfill an individual user's data request, becoming available for another user immediately after execution.

Such applications must manage user-access permissions (privileges). Permissions are typically not assigned to users directly. Instead, one defines "Roles" (e.g., primary data entry, protocol designer, study administrator) that define permissions (e.g., no access, read-only, read-write) with respect to various data components. Individual users are then assigned (or de-assigned) one or more roles. This indirect approach is more efficient: roles act as permission-setting shortcuts, and they are much fewer than users.

RDBMSs can be used to define roles: service account privileges are defined mostly at the RDBMS level. However, it makes sense to additionally define them at the application level - e.g., for study-level access, where a user is limited to accessing only one or two studies in a system. Application-level roles can be used to customize the user interface dynamically- e.g., by disabling menus or other user-interface objects that do not apply to the current user.

Many users tend to have similar roles across systems: permissions across systems must therefore be coordinated. Study-level access, for example, must always propagate across all systems used to manage study workflow.

#### 4.3.2 Maintaining Audit Trails: Restricting User Actions

High-end systems involving human subjects must maintain audit trails: audit-trail records are stamped with the ID of the user who made a change, and a date-time of change. When two systems interoperate, individual users' actions may often change data on both systems. Here, the originating system typically maintains the trail. If, however, the destination system is also required to log changes, then user identification and credentials (role information) must be transmitted - without requiring them to log on to the other system.

Transmitting user credentials also serves another purpose. It acts as insurance against buggy or malicious application code that attempts to execute operations on the second system that might exceed a particular user's authority, thus forestalling "privilege-escalation" attacks [27].

#### 4.3.3 The Need for Integrated Role Management: Single Sign-On

As the number of inter-operating systems grows, a unified approach to user/role management becomes essential. One widely-used approach is "single sign-on": rather than logging on to multiple applications individually, the user logs on to a single "authentication server" system which accesses a database of user-role information across applications, and transmits an encrypted token ("ticket") to an invoked application, which then authenticates the user and ascertains the user's privileges for that application. The framework we have devised is based on the Amazon Web Services algorithm description [28] and a schema published by Sheriff [29]. The schema and algorithm are described in detail in Additional file 2.

## 5 Conclusions

For management of longitudinal clinical studies involving biospecimen collection and analysis, integration of the capabilities of a CRIS and BDMS can offer significant benefits in terms of spectrum of functionality. Such integration is easier with open architectures and open-source designs or components, and we hope that our description of our own work will guide others in their efforts.

## 6 Availability and Requirements

We provide a design that can be used by investigators for their own purposes through a detailed technical description in the additional files associated with this paper.

## 7 Competing interests

The authors declare that they have no competing interests.

## 8 Authors' contributions

PMN implemented the software; RK and CRP determined the system's requirements. All three authors contributed to the writing of the paper. All authors read and approved the final manuscript

## Supplementary Material

Additional file 1 **Schema documentation**. Annotated description of the BDMS schema, and schema for role management and user authentication. Click here for file

Additional file 2 **Microsoft Access Schema**. Microsoft Access database containing the above schemas. TrialDB, the CRIS whose use is summarized in the paper, is freely available for downloading via http://ycmi.med.yale.edu/trialDB/open_source.shtm. Requirements: It requires an Oracle back-end schema (a SQL Server version is also available), the use of Windows 7 servers and Windows XP or Win 7 clients (for study design) and Internet Explorer v7 or later for the web browser. Detailed installation instructions are available at http://ycmi.med.yale.edu/trialdbdownloads/Installation%20Instructions.htm. Click here for file
