# Supplementary material for: Leveraging a clinical research information system to assist biospecimen data and workflow management: a hybrid approach
Source: J Clin Bioinforma. 2011 Aug 25;1:22. doi: 10.1186/2043-9113-1-22 (PMC3174108; doi:10.1186/2043-9113-1-22)
Supplement: Additional file 1 — Schema documentation. Annotated description of the BDMS schema, and schema for role management and user authentication. [file 2043-9113-1-22-S1.DOC]

# Appendix: Details of Schema

In figures 1 and 2 below, bolded columns represent primary-key columns. Columns that connect to columns of other tables via “relationship” lines and an infinity (“many”) symbol next to them are foreign keys.


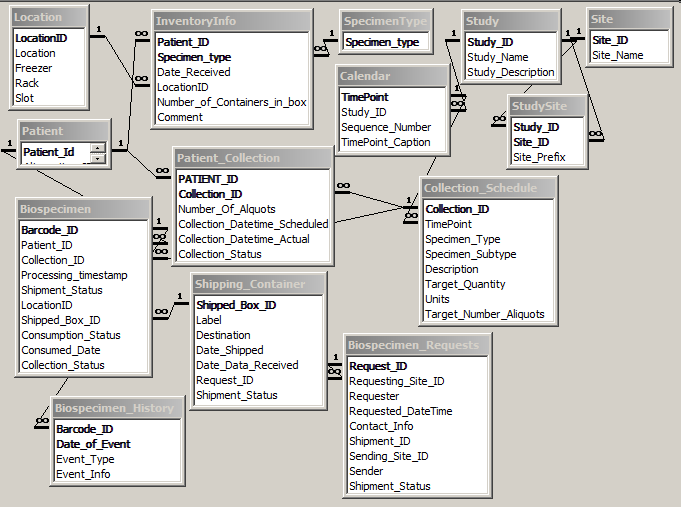


We emphasize that individual groups wishing to utilize the schema may need to modify it for their own purposes

## Metadata Tables

### Study

This table stores skeletal information on a study (protocol), imported from TrialDB: Study_ID (Identity, primary key), Study_Name (String), Study_Description (String)

### SpecimenType

Records a master list of specimen types across the system. The Specimen type may be as granular as needed. Thus, rather than recording the Specimen Type as “Blood” alone, one may make it as specific as needed (e.g., Blood/EDTA, Blood/Heparin,Serum). Columns: SpecimenType (string). If needed, a Specimen Type ID may be used as artificial primary key.

### Site

This table stores skeletal information on all research sites, imported from TrialDB:Site_ID (Identity, Primary key), Site_Name (String)

### StudySite

This many-to-many table records which sites participates in a given study. The primary key is (Study_ID, Site_ID ) both foreign keys into the previous two tables. The column Site_Prefix (String, optional), if not null, is used to compose an alphanumeric surrogate ID for a patient from a site.

### Calendar

This table stores study calendar metadata. gets information from TrialDB; only the bare minimum calendar information is imported: TimePoint (Identity, Primary key), Study_ID (Integer, foreign key into Study), Sequence Number (integer) and TimePoint_Caption (String)

### Collection_Schedule

This table is related many-to-one to Calendar: it records the details of specimen collection at each time point. Note that not all time-points will have entries in this table. Columns: Collection_ID (autonumber primary key), TimePoint (foreign key into Calendar), Specimen_Type, specimen subtype (optional- extra information if needed), Description (of specimen purpose), Target Quantity (numeric) and Units, target number of aliquots per specimen.

## Patient/Subject Related Imported Data Tables

### Patient

This table contains at least a unique Patient ID (primary key). Additional columns that identify a patient in greater detail are typically used, which vary depending on the installation.

### Biospecimen

This table records information on an individual collected biospecimen, as defined by the Patient, collection schedule information, and information related to the specific collection – e.g., whether collected, number of aliquots collected, date-time information.. A biospecimen is not associated with a barcode – the entities derived from it – aliquots, processed products – are. Note that:

- In some cases, a biospecimen will have a single derived item based on it.
- Storage information is recorded with the Subspecimen (derivative or aliquot), not with the biospecimen itself. While all subspecimens related to a biospecimen are generally stored together, this connection may be lost when subspecimens are shipped elsewhere, but not yet consumed.

Columns: Specimen_ID (artificial primary key), Patient ID (foreign key into Patient), Collection_ID (foreign key into Collection_Schedule), Number of aliquots collected, Scheduled Collection date-time, Actual collection date-time, Comments, Collection Status. The last is one of: Pending, Collected, Not Collected.

## Data Managed Primarily by BDMS

### Location

A master list of available storage locations: Columns: LocationID (artificial primary key), Location (e.g., building), Freezer. Rack, Slot. You may need to modify this table based on your needs.

### Subspecimen

This stores information on sub-specimens, which may be aliquots of the original specimen or processed products derived from a specimen. A subspecimen may in turn be derived from another subspecimen – e.g., a blood aliquot may be used to create DNA – so this table is related to itself (i.e., its records may represent an arbitrary hierarchy). Barcodes are associated with subspecimens, as is storage location information and consumption information.

Columns: Subspecimen ID (artificial primary key), Barcode ID Specimen ID (specimen of which this is an aliquot or derivative), Subspecimen Type (aliquot or derivative), Parent Subspecimen ID, (if derived from another subspecimen), Location ID (Storage Location), Shipment Status, Processing timestamp (when processed), Shipped Box ID, Consumption Status, Consumed Date..

### Biospecimen_History

This table serves as an audit trail for what happens to a given aliquot; records are generated and appended automatically. There is a record for every event (collection, shipping, processing, storage, consumption, etc.) Columns: Subspecimen_ID, Date of Event Event Type , Event Info. The last is text that depends on the Event – e.g., for storage, the storage location is recorded.

### Biospecimen_Requests

This table records requests made for a biospecimen. Columns: Request_ID (artificial primary key), Requesting_Site_ID, Sending_Site_ID (both foreign keys into Site), Requester (name), Requester Contact Information, Sender (name)., Destination (address)

### Shipping_Containers

This records shipments made against requests. There are one or more shipments for a request. Columns: Shipped_Box_ID (artificial primary key), Request_ID (foreign key into Biospecimen_Requests), Label, Destination, Date Shipped, Date Received, Shipment Status (one of: pending, shipping, received, rejected)

# Appendix 2: Implementing User Permissions with Single Sign-On


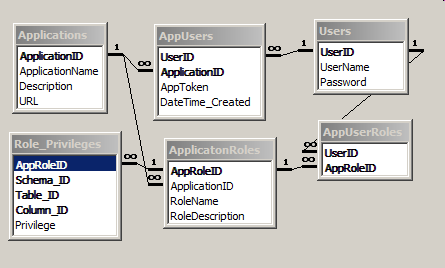


## Description of Tables

In figure 2 above, bolded columns represent primary-key columns. Columns that connect to columns of other tables via “relationship” lines are foreign keys.

### Users:

List of all users in an installation. Columns: UserID (primary Key), (Login)Username, Password (stored encrypted). Based on your needs, you may record additional identifying information here.

### Applications:

List of all applications available to an installation Columns: ApplicationID (primary key), Application Name, Description, URL to invoke application..

### ApplicationRoles:

A list of all named roles across all applications. Some of these may be imported from the DBMS that hosts an application’s data, others are imported from tables that an application may use to record role information. Columns are: AppRoleID (primary key), ApplicationID (foreign key into Applications), Role Name, Role Description.

### AppUsers:

This table does double duty. It records which users have permissions to use which application. It is also used to store a token (a 128-bit machine-generated unique identifier) against a UserID/ApplicationID row, along with a time-stamp (using Universal Coordinated Time (UTC)– same as Greenwich Mean Time) accurate to the nearest millisecond, using the computer clock.

Columns:: UserID, ApplicationID (both foreign keys into Users and Applications respectively), Apptoken (contains a temporarily created UID), and DateTime_Created (records date/time of UID creation).

### AppUserRoles:

Records which user has which roles. Columns: UserID (foreign key into Users), AppRoleID (foreign key into ApplicationRoles)

### Role_Privileges

This table records what access privileges a given role has for an element in a database schema. An element is defined by a combination of a database schema, a table within the schema, and (optionally) a column within that table. If the last is not specified, the privilege specified applies to all columns in that table.

In the design described here, we use a set of metadata tables (schema, table_info, column_info) containing information on all database schemas used across applications; all of these tables utilize artificial primary keys, so that in the Role_Privileges table, we use Schema ID, Table ID, and Column_ID.

Columns: AppRoleID (foreign key into ApplicationRoles), Schema ID, Table ID, Column ID, Privilege. The last is one of: read, change, add, delete. Add and Delete apply only to the entire table (i.e., the ability to create or delete rows). Read and Modify apply to individual columns.

If no entry exists for a role against a given Schema/Table pair, it means that even read access to that table is not permitted.

### Additional Tables

Based on your needs, you may incorporate additional tables for audit trail purposes. For example, you may use tables to record the date/times when a user was added/removed, when access to an application was granted/revoked for a given user, and when application roles were granted/revoked for individual users.

## User Authentication /Role Discovery via Single Sign-On

The process is as follows:

1. The user logs in to the authentication server via a web page using the https protocol (to prevent network-based electronic eavesdropping).
2. The server application encrypts the password and checks for the existence of the user-name/encrypted-password combination in the Users table using a stored procedure. If the combination exists, the user ID is fetched. Otherwise, login fails.
3. The server now searches AppUsers to determine what applications the user can access, and presents these. The user selects an application to invoke.
4. The server now determines the current date/time (as UTC), generates a random global unique identifier (GUID) and stores these in the columns (Datetime_Created and AppToken) of the row corresponding to the UserID/ApplicationID pair..The GUID is typically 128 bits long.
5. The server now appends the GUID to the datetime, and computes a cryptographic hash (a message digest) of the result. (Standard hash functions such as SHA-1 can be used.) The server now transmits the message digest, along with the application ID and User ID (the latter two as plaintext). This message is sent over the network to the target application’s URL: this application resides on a separate machine, maybe
6. The target application uses the UserID/Application ID pair to look up the AppUsers table, and fetches the GUID and Datetime. Using the same cryptographic algorithm, it computes a hash of the datetime/GUID combination and compares these to the transmitted hash. If these are identical, it means that the message the application received originated from the authentication server, and not somewhere else (e.g., an intruder attack).
7. The target application promptly erases the GUID and datetime for the given user/application pair in the AppUsers table; this ensures that the GUID has “expired”.
8. The target application may also check that the date/time it receives a message (as measured by the system clock), minus the date/time when the message originated (as initially looked up in the AppUsers table), does not exceed a certain preset time limit. (Depending on the scenario, this limit may be a few seconds, or even up to 15 minutes if this scenario executes over the Internet). If this limit is exceeded, the message is treated as “expired”.
9. Given the UserID/ApplicationID pair, the target application looks up the roles for the user in AppUserRoles, and accesses Role_Privileges to get information that can be used to tailor system behavior (e.g., the user interface).
